# Challenges and recommendations to increasing the use of exome sequencing and whole genome sequencing for diagnosing rare diseases in Brazil: an expert perspective

**DOI:** 10.1186/s12939-022-01809-y

**Published:** 2023-01-13

**Authors:** Têmis Maria Félix, Carolina Fischinger Moura de Souza, João Bosco Oliveira, Mariana Rico-Restrepo, Edmar Zanoteli, Mayana Zatz, Roberto Giugliani

**Affiliations:** 1 Medical Genetics Service, Hospital de Clinicas de Porto Alegre, Rua Ramiro Barcelos, 2350, Porto Alegre, 90,035–903 Brazil; 2 Hospital Israelita Albert Einstein, São Paulo, Brazil; 3 Americas Health Foundation, Bogota, Colombia; 4 Faculdade de Medicina, Universidade de São Paulo (FMUSP), São Paulo, Brazil; 5 Human Genome and Stem-cell Research Center, Institute of Biosciences, University of São Paulo, São Paulo, Brazil; 6 House of Rares, Porto Alegre, Rio Grande do Sul Brazil

**Keywords:** Brazil, Rare diseases, Diagnosis, Exome sequencing, Screening, Whole genome sequencing, Genomics

## Abstract

Early diagnosis of genetic rare diseases is an unmet need in Brazil, where an estimated 10–13 million people live with these conditions. Increased use of chromosome microarray assays, exome sequencing, and whole genome sequencing as first-tier testing techniques in suitable indications can shorten the diagnostic odyssey, eliminate unnecessary tests, procedures, and treatments, and lower healthcare expenditures. A selected panel of Brazilian experts in fields related to rare diseases was provided with a series of relevant questions to address before a multi-day conference. Within this conference, each narrative was discussed and edited through numerous rounds of discussion until agreement was achieved. The widespread adoption of exome sequencing and whole genome sequencing in Brazil is limited by various factors: cost and lack of funding, reimbursement, awareness and education, specialist shortages, and policy issues. To reduce the burden of rare diseases and increase early diagnosis, the Brazilian healthcare authorities/government must address the barriers to equitable access to early diagnostic methods for these conditions. Recommendations are provided, including broadening approved testing indications, increasing awareness and education efforts, increasing specialist training opportunities, and ensuring sufficient funding for genetic testing.

**Supplementary Information:**

The online version contains supplementary material available at 10.1186/s12939-022-01809-y.

## Introduction

The Brazilian Ministry of Health (MoH) defines a rare disease (RD) as affecting 65 or fewer per 100,000 individuals, following the World Health Organization (WHO) definition [1]. Global RD prevalence is estimated to be 3.5–5.9%, affecting 263–446 million people [2]. Brazil has 213 million inhabitants, with an estimated 10–13 million people living with a RD [2]. Despite the considerable heterogeneity between RDs, some common characteristics are their chronicity, severity, and early onset. Some are degenerative and fatal. They profoundly affect quality of life (QoL) and compromise autonomy, generating a substantial psychosocial burden on patients and their families. Around 6172–9603 unique RDs exist, of which 60–72% are genetic [2, 3]. Most have pediatric onset (69.9%), followed by both pediatric or adult onset (18.2%), and exclusively adult onset (11.9%). Genetic RDs are a leading cause of death in children less than 10 years of age [4] and are the leading cause of mortality and morbidity in neonatal and pediatric intensive care units (ICUs) in the United States (US) [5]. In Brazil, congenital disabilities were the second cause of death in children under five in 2017 [6].

Early genetic diagnosis for patients with RDs allows costly and unnecessary procedures, tests, and medical appointments to be avoided. Identifying the causative molecular basis of RDs allows an etiologic diagnosis, which can trigger a chain reaction of health outcome-altering events, such as pharmacotherapy changes, specialist referral, avoidance of unnecessary procedures or treatment, ineffective treatment suspension, and palliative care initiation. Patients with RD usually face a “diagnostic odyssey,” which lasts many years and has a high cost to caregivers, healthcare systems, and society [7]. A study on mucopolysaccharidosis found a 4.8-year delay from symptom onset until diagnosis [8]. Without a diagnosis and appropriate therapy, many patients face severe adverse events, sometimes including admission to ICU [3]. These diagnostic delays have repercussions on the health and economic burden created by RDs and continue to represent an unmet need in Brazil. To better understand the current landscape of genetic and genomic testing for RDs in Brazil, we convened an expert panel to assess the landscape, identify barriers and opportunities for improvement, and propose solutions.

## Methods

Americas Health Foundation (AHF) identified six experts in RDs, including medical geneticists, pathologists, neurologists, and biologists from Brazil. They were convened for a three-day virtual meeting to develop recommendations to increase the use of ES and WGS for diagnosing RDs in Brazil. AHF conducted a search using PubMed, MEDLINE, and EMBASE to identify physicians who have published on RDs since 2017. Augmenting this search, AHF contacted opinion leaders from LA to corroborate that the list of individuals adequately represented the field. All experts who attended the meeting are named authors of this manuscript. An AHF staff member moderated the discussion. The authors retain complete control over the content of the paper.

**Table:** 

Search strategy: AHF conducted a literature review using PubMed, MEDLINE, and EMBASE for any publications on early diagnosis of RDs. The following search terms were used: “rare diseases,” “diagnosing rare diseases,” “exome sequencing,” “rare disease screening,” and “whole genome sequencing” in combination with “Brazil” from 01/01/2017 to 09/06/2021. The articles found were in English, Portuguese, and Spanish. Particular attention was paid to identifying literature and research from LA	

AHF developed specific questions, based on the aforementioned literature search, to address the challenges in diagnosing RDs in Brazil and assigned one to each expert. (Supplementary Material 1) A written response to each question was drafted by each expert based on the literature review and personal expertise. The entire panel reviewed and edited each narrative during the three-day conference through numerous rounds of discussion until total agreement was reached. When the panelists disagreed, additional dialogues took place until all panel members agreed on the content included in this document. The developed recommendations were based on the available literature, expert opinion, and personal experience and were approved by the entire panel. After the conference, the final manuscript was distributed by email to the panel for review and approval.

### Current state of ES and WGS in Brazil/ literature review

#### Genetic testing and molecular diagnosis of RDs

Genetic RDs are caused by chromosomal anomalies or single or multiple gene mutations. Traditionally, molecular diagnosis has been performed using serial tests based on differential diagnosis or hypothesis with single-gene analysis, newborn screening panels, metabolic testing, cytogenetics, and gene panels associated with specific disease types, among others. The chromosome microarray (CMA) analyzes copy number variations (CNV) greater than 400 kilobases (kb) of DNA. It was recommended as the first-line genomic test for unexplained intellectual disability, autism spectrum disorders, or congenital anomalies, according to the American College of Medical Genetics and Genomics (ACMG) and the American Pediatric Association (APA) [9–11]. However, a diagnosis is not reached using CMA in 80–85% of these cases [12]. This limitation instigated a paradigm shift to the use of exome sequencing (ES) and whole genome sequencing (WGS), because their potential to reduce the “diagnostic odyssey” has been demonstrated in situations such as critically ill children, developmental delays, and congenital anomalies [13–15]. By allowing simultaneous examination of several or all genes, ES and WGS have the potential to achieve an etiologic diagnosis in time to influence acute management and open doors for precision medicine in RD. This ability is being evaluated by the medical community and healthcare providers in Brazil; however, several examples of its benefits are already available in severely ill children [15, 16].

ES targets all exons and canonical splice sites of ~ 20,000 genes accounting for 2% of the genome [17]. It provides a diagnostic yield of approximately 25% for intellectual disabilities and congenital anomalies. However, it has limitations because it does not detect small CNV (< 5 kb), insertion-deletions (indels), intronic single nucleotide variants (SNV), or complex structural genomic variations [12].

Unlike ES, WGS offers the potential of a single test that captures nearly all genomic variations in an unbiased manner, evaluating approximately 92% of the genome [18]. The clinical utility of WGS is demonstrated primarily through its superior diagnostic performance, allowing patients to end a diagnostic odyssey or prevent it all together when implemented earlier in the diagnostic pathway [19].

A meta-analysis comparing ES, WGS, and CMA for molecular diagnosis of children with suspected genetic disorders showed a diagnostic yield of WGS of 41%, ES of 36%, and CMA of 10%, suggesting that ES and WGS should be the first-line genomic test for children [4]. Another study comparing CMA and WGS in 100 pediatric patients with congenital anomalies and neurodevelopmental disorders found that CMA had a diagnostic rate of 8%, adding target gene sequencing to CMA increased the rate to 13%, while WGS had a rate of 34%. A similar study showed that CMA or gene panel-based testing yielded 24% and WGS 41% [19]. Prospective data provides new insights into how WGS could transform genetic assessment in pediatrics and RD. As clinical WGS becomes feasible on a larger scale, it may become a first-tier diagnostic test. Hypothesis-driven testing would still be performed, by limiting the initial analysis of WGS data to specific genes and loci. Provided coverage is adequate; this would allow the clinician to retain control over the scope of testing while facilitating future comprehensive interrogation of the genome. Further research will help delineate the potential advantages and limitations of such an approach [20]. It should be noted that CMA, ES, and WGS have limitations in detecting epigenetic and dynamic mutations and complex multifactorial genetic disorders [20].

Rapid whole genome sequencing (r-WGS), which does not have methodological differences but offers a faster turnaround time for analysis, has been used to evaluate severely ill children in neonatal or pediatric care units. This population requires prompt diagnosis and intervention; r-WGS can provide results in 26–48 hours. A cohort of acutely ill infants showed that the diagnostic yield of r-WGS was 43% compared to a conventional genetic test of 10% [21]. This rapid diagnosis allowed changes in clinical or surgical treatment, avoiding morbidity, and reducing acute mortality, thus reducing hospitalization costs. Several studies showed similar results for severely ill children [15, 22–24].

#### Economic considerations of ES and WGS implementation

Early genetic diagnosis for patients with RDs diagnosis is essential to avoid unnecessary management and guide early change in treatment, impacting the cost burden exerted on the healthcare system in the time elapsed before a patient receives a diagnosis. The logistics of widespread testing are not complicated. Samples can be shipped from rural areas to centralized laboratories, minimizing existing geographical disparities and additional expenses incurred by patients living in smaller cities.

Some studies have demonstrated the ability of ES and WGS to increase the rate of genetic diagnosis and reduce economic costs compared to traditional genetic testing [13, 25]. In one study, ES achieved a molecular diagnosis in 52% of children suspected of having a monogenic condition but who had not had any single-gene or panel testing. In 35% the diagnoses were unexpected, and clinical management was altered in 26%.These findings resulted in a considerable reduction in economic costs compared to traditional testing methods. This cost reduction was more evident when ES was the first diagnostic approach [26]. Therefore, ES would likely be more cost-effective if used earlier in the diagnostic pathway (after CMA fails to diagnose) for individuals with unexplained developmental disabilities and multiple congenital anomalies [13]. A recent study evaluated the cost-effectiveness of early ES in pediatric patients with complex monogenic conditions compared to a matched historical cohort. In addition to finding increased diagnostic yield, singleton/ES significantly reduced costs compared to a conventional investigation [27].

While no studies have examined the cost-effectiveness of early ES usage against standard genetic condition inquiry in Brazil, its early application for monogenic illnesses may have comparable local impacts. Project Baby Bear showed that implementing rapid precision medicine providing r-WGS for children under 1 year and within 1 week of hospitalization improves outcomes and reduces costs. In the study of 184 severely ill children, 40% received a diagnosis 3 days after admission. The combined testing and precision medicine cost was US$1.7 million; however, savings reached US$2.2–2.9 million in medical care [15]. Although ES and WGS are considered cost-effective in other regions, it is essential to determine the cost to the healthcare system if offered to the Brazilian population. Pricing of ES may be the most critical aspect to be considered to ensure the sustainability of the technology for Brazil’s national healthcare system (SUS), the hospitals offering it, and the payers.

#### Genetic testing for RD: Brazilian landscape

The organized care of patients with RD within the Brazilian public healthcare system began in 2014 when the Brazilian MoH established resolution 199/2014 for the Brazilian Policy of Comprehensive Care for People with Rare Diseases (BPCCPRD). This initiative aims to reduce morbidity and mortality and secondary manifestations while improving the QoL of patients with RD through promotion, prevention, and early detection activities. The resolution also aims to increase the opportunity to receive adequate treatment, prevent disability, and promote palliative care. Under the BPCCPRD, care for RDs should be provided through the Brazilian Unified Health System (*Sistema Único de Saúde* or SUS) and the establishment of two RD centers: Rare Disease Specialized Care Centers and Rare Disease Reference Centers, which will both be referred to in this paper as RDRC. They differ in complexity, with the latter being more comprehensive and requiring medical geneticists and other medical specialists on staff. These RDRCs have access to additional funding and may order biochemical assays, multiplex ligation probe amplification, CMA, and ES (to a limited extent), often not available to the rest of the public healthcare system. This resolution was enacted in late 2016 when the MoH designated the first RDRC in the country.

##### Unified health system (SUS)

In addition to the 19 RDRCs, primarily located in large cities, numerous academic institutions also treat patients with RDs in the SUS. Although all Brazilian citizens have coverage under SUS, access to genetic testing is quite difficult. The situation has improved with the implementation of BPCCPRD [28], which included the “mutation identification by sequencing of an amplicon up to 500 base pairs” in the procedure list covered by SUS [29]. This policy opened the first door in SUS for DNA sequencing-based tests. A major advance occurred in 2020. After the positive appraisal from CONITEC (Health Technology Assessment Agency for SUS), the MoH Resolution 1111/2020 included ES coverage for SUS to investigate intellectual disability of unknown cause with no age limit [30]. However, since then, no specific reimbursement or payment has been linked to ES, generating a significant barrier to the adoption of the test since public providers receive no incremental reimbursement for it.

##### Private healthcare

The National Agency of Supplementary Health (ANS) regulates the test procedures and tests that all private healthcare policies in Brazil should cover. As of December 2021, 23% of the Brazilian population has access to private healthcare coverage [3]. ANS began to include genetic tests in its portfolio in 2014 under *Note 876.GGRAS/DIPRO/ANS* [31], enabled mandatory coverage of genetic tests for 22 conditions. Resolution 465/2021, which expanded the portfolio of genetic tests to include the coverage of 47 genetic indications, included ES and approved its use in the private healthcare system only for the investigation of intellectual disabilities of unknown origin after a negative CMA test [32]. Despite being included in the mandatory list for private coverage, genetic tests in private healthcare are subject to Technical Guidelines (DUT) number 110, which creates significant barriers for patients and healthcare professionals to access genetic tests. The 67 pages of the *DUT #110* contain several technical requirements for coverage, and most providers and private healthcare policies, prefer not to request or pay for genetic tests given the enormous complexity of requesting them, according to DUT #110.

The approved use of ES is only for intellectual disability, given the scientific evidence for the high diagnostic yield of ES and WGS in different clinical scenarios [16, 33, 34]. Table 1 summarizes the approved indications for CMA, ES, and WGS through private and public healthcare in Brazil as of publication. Table 2 provides a list of indications that would benefit from the approval of these technologies.

**Table 1:** Approved CMA, ES, and WGS testing for RD in Brazil’s public and private healthcare setting

APPROVED INDICATIONS	PUBLIC SYSTEM (SUS)			PRIVATE HEALTH INSURANCE		
	CMA	ES	WGS	CMA	ES	WGS
Intellectual disability	✓	✓		✓^a^	✓^b^	
Developmental delay	✓			✓^a^	✓^b^	
Multiple congenital anomalies	✓			✓^a^		

^a^Only after a negative karyotype

^b^Only after negative CMA

*CMA* Chromosomal microarray analysis, *ES* Exome sequencing, *WGS* Whole genome sequencing, *SUS* Sistema Único de Saúde

**Table 2:** Indications for CMA, ES, and WGS that would benefit from approval in Brazil

UNAPPROVED INDICATIONS FOR CMA, ES, AND WGS THAT WOULD BENEFIT FROM APPROVAL	
Critically ill children	Neurodegenerative diseases
Epilepsy	RD suspicion based on family history or clinical presentation
Cardiogenetic diseases	Hereditary cancers
Autism spectrum disorders	Undiagnosed diseases
Inborn errors of immunity	

*CMA* Chromosomal microarray analysis, *ES* Exome sequencing, *WGS* Whole genome sequencing

## Current ES and WGS in Brazil

Molecular testing for some genetic disorders such as neuromuscular disorders, developmental disorders, inborn errors of metabolism, and intellectual impairment, among others, has been offered by several laboratories in Brazil since 2000. NGS was launched in Brazil around 2010, opening the opportunity for molecular diagnosis through targeted gene sequencing panels directed at a group of disorders, such as neuromuscular disorders and inherited forms of cancer or skeletal dysplasia. These panels may be helpful when clinical data suggest a specific diagnosis. They are less expensive and can focus on the suspected disease without the implications of ethical issues related to possible incidental findings with expanded sequencing.

Several laboratories in Brazil now offer ES, as this technology has a lower cost than WGS. However, these are primarily private laboratories in which SUS does not provide coverage. Therefore, access is restricted to those who can pay or have private insurance. In Brazil, WGS was used almost exclusively in research settings, such as universities or research institutions, but it has just begun to be offered in limited clinical settings through private laboratories.

## Challenges to ES and WGS adoption in Brazil

Adopting ES and WGS requires complex equipment and trained personnel for data interpretation and application in the clinical setting. Substantial challenges arise from complex regulatory hurdles, costs, and infrastructure needs. The main challenges identified by this panel are described below.

### Geographical maldistribution of resources

Geographical disparities in access to and quality of healthcare permeate Brazil, a situation aggravated by the complexity required for RD care. Most RDRCs, especially the best equipped, are in the South and Southeastern regions, with a much higher concentration of medical facilities and specialists. The North, Central-West and Northeast regions have proportionally fewer facilities and professionals [35]. 20% of the Brazilian population lives in rural areas or small cities with less than 5000 inhabitants and minimal resources [36]. In these rural settings, healthcare is provided primarily by “Family Health” teams, which operate under the “Primary Health Care” policy and are generally not sufficiently trained to recognize and suspect RD. Even when a RD is suspected and referral to a specialized service is requested, access delays may last many years and can even be more severe when RDRCs are not in the same state as the patient [37].

Establishing diagnostic networks, which allow institutions without laboratory facilities to have free real-time access to expert assistance and specialized laboratory support, was one solution to mitigate resource maldistribution. This strategy was implemented in the 2000s with the Information Service on Inborn Errors of Metabolism (SIEM) and was expanded to include laboratory diagnostic support after 2004 [7, 38]. This diagnostic support system has been widely used to achieve biochemical diagnosis and allows the sequencing of single genes or small gene panels. This program does not currently support ES or WGS but could also be implemented for this type of testing.

Some additional initiatives are contributing to referral optimization in underserved areas. For example, ‘Telessaude’ prioritizes cases that require urgent evaluation and is available in some southern states such as Sao Paulo and Rio Grande do Sul [39]. During the COVID-19 pandemic, this was one of the critical conduits for the early examination of genetic abnormalities. Another initiative focused on RD is the program “Hello Genetics,” which is intended to provide a free helpline to healthcare professionals who suspect a genetic disease in the primary care setting [38].

### Reimbursement

In practice, genetic testing is not accessible to most of those who require it in Brazil. Although there has been an increase in policy coverage for ES testing in SUS and private healthcare, reimbursement remains a practical issue, and coverage is restricted to only one indication, intellectual disabilities. Although reimbursement in the private sector is adequate, it represents a major barrier in the public sector. The actual costs of implementing this technology exceed the reimbursement assigned, making testing unsustainable and hindering adoption. The RDRC only has R$800.00 (~US$ 142.00) for coverage per patient every 3 months for any molecular testing. This amount is insufficient for complex testing, such as ES, WGS, or CMA. Even in centers with the necessary equipment for in-house testing, the service is not sustainable with current reimbursement.

### Cost

Most challenges in implementing ES and WGS in Brazil are due to a lack of funding for the public healthcare system. These technologies require a robust infrastructure of human, technological, and bioinformatic resources that most institutions in Brazil lack and for which substantial investments are needed. Thus, most RDRCs outsource their testing to private laboratories. In addition, only 19 RDRCs are established in the country, which is insufficient to meet demand. While testing costs have begun to decrease in the country, an average clinical ES-based genetic test in Brazil costs about 3–4 times the minimum wage, the highest income tier for a third of the population [36].

### Awareness and education

There is a general lack of education about ES and WGS among all stakeholders about the importance of a diagnosis to patients, families, healthcare systems, and society. There is limited medical education and awareness regarding the value of this technology among the medical community (i.e., the actual tests, patient selection, clinical utility, and reimbursement practices), with a large knowledge gap between physicians at specialized and academic centers and those at the primary setting. Notably, a significant additional barrier to broad adoption is a general lack of guidance and standards on interpreting the results, clinical implementation, and uncertainty about possible ethical implications. Suboptimal training and deviation from national guidelines often lead to quality issues in the reports [40].

### Specialist shortage

ES and WGS require highly trained healthcare professionals in genetic counseling, medical genetics, bioinformatics, and laboratory sciences. In Brazil, there is a severe shortage of these specialists which hinders technology implementation. Adequate local training programs are minimal, and genomics training is not included in medical school curricula. Furthermore, most specialists are concentrated in the main cities, creating access disparities.

### Paucity of local data

Although several initiatives attempt to characterize the Brazilian population’s genomic landscape, data remains scarce. This scarcity represents a technical barrier to analyzing and interpreting the data generated from ES and WGS. Specifically, in the context of genetic testing, the availability of large-scale genetic data in Brazil would facilitate variant classification, reducing the number of variants classified as of unknown significance. It would also contribute to understanding the pathogenicity of different genomic variants in the Brazilian population.

## Ongoing initiatives

The MoH has recently spurred an initiative called the Brazilian Genomes Project, which aims to sequence the whole genomes of up to 100,000 people in the next 5 years. There are currently three pilot projects under this initiative: a population genomics project (DNA of Brazil), a RD genomics project (Rare Genomes Project), and a cardiovascular genomic project (RENOMICA). The Rare Genomes Project is an Albert Einstein Jewish Hospital initiative in Sao Paulo in partnership with the MoH [41]. It began in 2020, aiming to sequence over 8000 patients with RD seen in the Brazilian public health system before the end of 2023. These patients are recruited from participating centers throughout the country. By February 2022, more than 4179 probands and 4687 relatives were recruited within 18 predefined disease groups, and WGS was performed in over 3350 patients. Preliminary results demonstrate a high diagnostic rate (35.7%), with 13% of those findings being variants difficult to identify by panels or ES. In addition, the samples are being stored in a biobank to accelerate future research in the field. This project will allow the creation of the largest Brazilian WGS database of patients with RD and provide subsidies to assist in implementing genetic tools for future use in the public healthcare system.

Another example is the Brazilian Initiative on Precision Medicine, established to generate genomic data of the admixed Brazilian population [42]. This program remains active, making the data publicly available. In addition to exomes and CMA, genomic and other omics data will soon be available. The project currently has nine databases, including reference population and diseases, with information on 948 individuals.

The 80 Plus Project was launched in 2008 by HUGH-CEL, including the SABE (Health, Well-being, and Aging), a genomic census-based cohort of about 1400 Brazilians aged 60 years and older with a follow-up since 2000. The aims were to have a reference genomic database for the Brazilian population and contribute to understanding the factors related to lifespan [43]. In the first phase, ES was performed on 609 SABE individuals. More than 200,000 novel variants identified were absent from major public databases. More recently, the HUGH-CEL group has completed the WGS of a cohort of 1171 elderly individuals. It is the largest cohort of WGS of elderly individuals in Latin America [44]. Interestingly, about two million genetic variants not represented in international databanks have been identified, illustrating the importance of studying admixed populations [45]. More recently, this group has performed ES in a recently established COVID-19-recovered group of nonagenarians and centenarians [46].

A pilot project, the “Odyssey Project,” which will perform r-WGS on 100 critically ill babies (and their parents), is also underway. It is supported by a private genomics laboratory and involves at least two leading Brazilian institutions (Hospital de Clinicas de Porto Alegre and Instituto da Criança-USP) (DASA).

## Conclusions

There is no doubt that achieving an early diagnosis of RD and reducing the length of the diagnostic odyssey benefits everyone: the patient, family members, healthcare professionals, healthcare systems, and society. Obtaining an earlier, more accurate, and faster genetic diagnosis is essential to offering patients the best medical care and effective genetic counseling. ES and WGS can effectively detect a range of pathogenic variants that may inform management decisions, yielding clinical value for the patients and their families and potential economic value for healthcare systems. While there is evidence of the high burden of RD on patients, relatives, and the healthcare system, the exact financial impact is difficult to estimate in Brazil [45, 47].

**Fig. 1:**
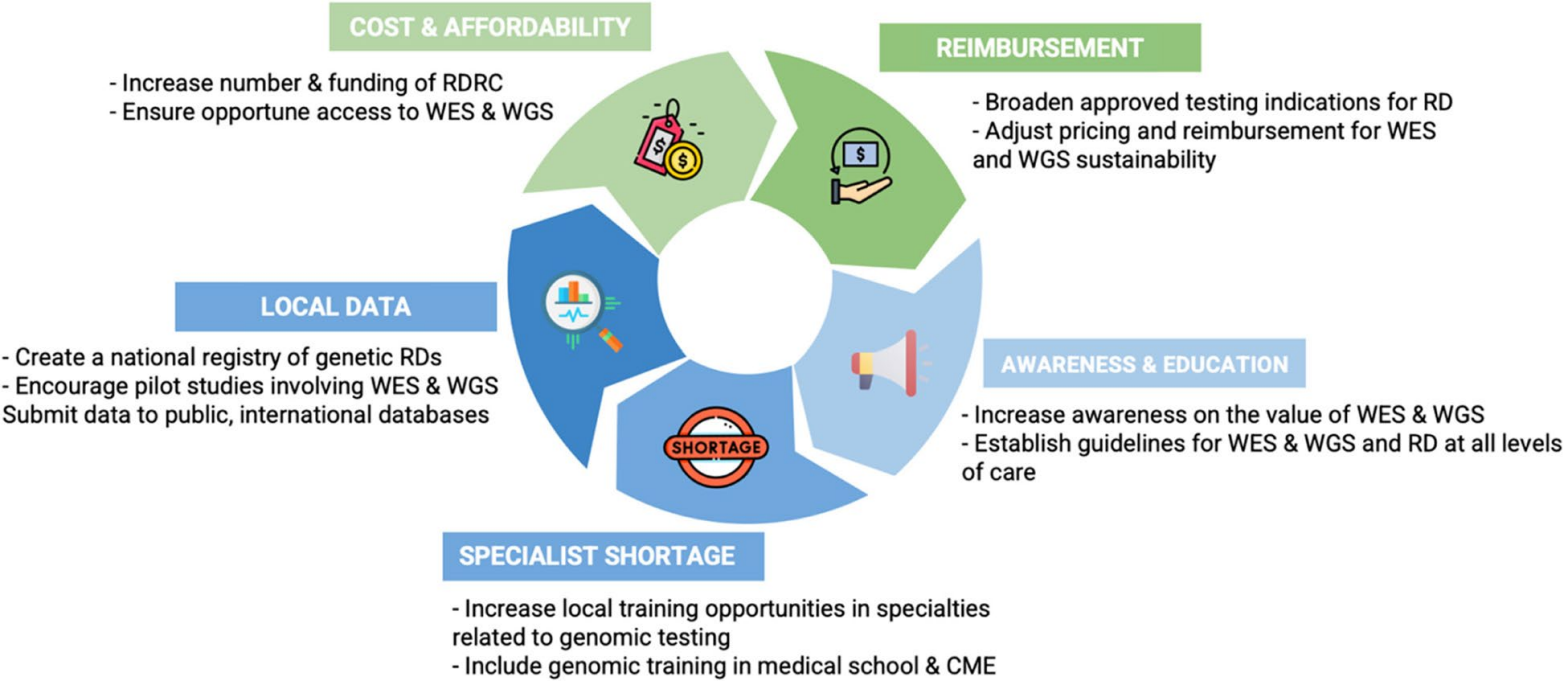
Challenges and recommendations for the widespread adoption of ES & WGS for RDs in Brazil Legend: RDRC: rare disease reference centers; ES: exome sequencing; WGS: whole genome sequencing; RD: rare disease; CME: continued medical education

To advance care and reduce the burden of RDs, the healthcare authorities and government must ensure equitable access to the best diagnostic methods, including ES and WGS, for those who would benefit from them. Globally, genome sequencing has been deemed as first-line testing in many clinical situations as a promising new technology to improve future medical management. Although the obstacles that challenge the adoption of ES and WGS for RD, Brazil could emerge as a model for achieving the opportunities this technology offers in Latin America. To this end, the following recommendations are proposed with the stakeholder(s) responsible for the change. Figure 1 summarizes the challenges identified and proposed solutions to increase the adoption of ES and WGS for RDs in Brazil. These may apply to other limited-resources settings with similar issues.

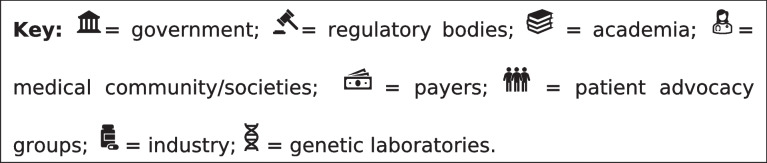



### Increase access and availability of testing by

- Increasing the number of designated RDRCs throughout the country and provide sufficient funding for existing RDRCs to establish quality infrastructure for testing
- Broadening current indications both in the private and public systems to include coverage for other clinical conditions where ES and WGS have shown high diagnostic efficacy, such as developmental delays, congenital anomalies, critically ill children, neurodegenerative diseases, epilepsy, RD suspicion based on clinical presentation or family history, cardiogenetic diseases, autism spectrum disorders, inborn errors of immunity, undiagnosed diseases (See Table 2) [16, 33, 48, 49].
- Ensuring opportune access to accurate ES and WGS, treatment if available, genetic counseling, and RD management with well-trained specialists who also follow ethical precepts in conducting the investigation and follow-up of the patient and their family
- Establishing designated pricing and reimbursement for ES and WGS that is coherent with the actual cost of implementing and using this technology to ensure its sustainability for those who provide it

### Create education and awareness on ES and WGS by

- Bringing together stakeholders to advocate for increased awareness about the value of ES and WGS in the diagnosis and management of RD in Brazil [50]
- Increasing local training opportunities for medical genetics, genetic counseling, laboratory skills, and bioinformatics to increase the availability of these healthcare professionals in the country
- Include genomics training in medical school curricula and implement continuing medical education programs for specialists

### Develop clinical practice guidelines to

- Train the primary care level to suspect RD and understand appropriate referral situations
- Establish diagnostic protocols based on clinical signs and symptoms for the use of ES and WGS as first-tier tests in the diagnostic pathway
- Implement and enforce guidelines and enact mandatory certification for molecular testing laboratories regarding result interpretation and practical clinical implementation to ensure quality control for genomic testing and application [51]
- Address the ethical implications of performing ES and WGS for patients with RD and train healthcare professionals on how to handle sensitive information

### Increase local data generation by

- Developing robust genomics initiatives to characterize Brazil’s genetic diversity, including a comprehensive registry of rare genetic diseases to understand specific epidemiologic aspects of genetic variation and frequencies
- Carry out pilot studies involving ES and WGS to understand and overcome implementation challenges for new technologies and establish the cost-effectiveness of genomic diagnosis for RD on which to base policy decision-making
- Submit assertions to international public databases to increase the representation of Brazilian data within these databases

## Supplementary Information

**Additional file 1: Supplementary Material 1.** Questions for panel response.

## Data Availability

Not applicable.

## Abbreviations

ACMG: American College of Medical Genetics and Genomics
AHF: Americas Health Foundation
ANS: National Agency of Supplementary Health
APA: American Pediatric Association
BPCCPRD: Brazilian Policy of Comprehensive Care for People with Rare Diseases
CMA: Chromosome microarray
CME: Continued medical education
CNV: Copy number variations
ES: Exome sequencing
ICU: Intensive care units
MoH: Ministry of Health
QoL: Quality of life
RD: Rare disease
RDs: Rare diseases
RDRC: Rare disease reference centers
SABE: Health, Well-being and Aging
SIEM: Service on Inborn Errors of Metabolism
SNV: Single nucleotide variants
US: United States
WGS: Whole genome sequencing
WHO: World Health Organization
