# Supplementary material for: Challenges and recommendations to increasing the use of exome sequencing and whole genome sequencing for diagnosing rare diseases in Brazil: an expert perspective
Source: Int J Equity Health. 2023 Jan 13;22:11. doi: 10.1186/s12939-022-01809-y (PMC9837951; doi:10.1186/s12939-022-01809-y)
Supplement: Supplementary file 1 — Additional file 1: Supplementary Material 1. Questions for panel response. [file 12939_2022_1809_MOESM1_ESM.docx]

**Supplementary Material 1. Questions for panel response.**

1. **Epidemiology and Burden of Disease**
   1. What is the clinical and economic burden of disease of rare undiagnosed genetic disorders (RUGD) in Brazil?
   2. What is exome sequencing (ES) and how does it differ from whole genome sequencing (WEG)?
   3. What clinical indications can benefit the most from these diagnostic tests?
2. **Diagnosis**
   1. How are patients with rare undiagnosed genetic disorders (RUGD) currently being diagnosed in Brazil? Which local, regional, or international clinical practice guidelines are followed and what do they recommend in terms of ES/WGS for rare diseases?
   2. How do ES/WGS impact the clinical management of rare genetic diseases for both patients and healthcare providers (i.e., diagnosis and treatment, early detection, quality of life, mortality)
3. **Access and Barriers**
   1. What is the status of availability, access, and use of whole exome/genome sequencing in Brazil? Does the Brazilian scenario differ from the global scenario?
   2. What are the barriers to the widespread adoption of ES/WGS in Brazil (i.e., awareness, infrastructure, human resources, HTA-DUT, adequate training, representation of Brazilians in public genomic reference databases)?
4. **Policy and Regulatory Aspects**
   1. What are the health policies surrounding ES/WGS for the public health system in terms of indications? (Which are approved and for which indications? Should these indications be expanded?) How do reimbursement codes in the public system impact access and availability of molecular testing, including whole exome/genome sequencing?
   2. In contrast, what are the policies surrounding ES/WGS for the private health system in terms of indications and coding? (Which are approved and for which indications? Should these indications be expanded?)
5. **Economic Considerations**
   1. Considering the efficacy of ES/WGS, can the use of WES/WGS have and economic impact on patient outcomes and long-term healthcare expenditure in Brazil? What should be considered in terms of pricing, contracting, and reimbursement to ensure the sustainability of the technology for SUS, the hospitals offering it, and the payers?
6. **Recommendations**
   1. What are recommendations for advancing the diagnosis and reducing the burden of rare diseases?
   2. Please provide a multi-stakeholder approach and specific recommendations for the public and private systems (physicians/medical societies, government/regulatory agencies, payers, hospitals/institutions)?
